# Comprehensive study reveals phenotypic heterogeneity in *Klebsiella pneumoniae* species complex isolates

**DOI:** 10.1038/s41598-024-55546-z

**Published:** 2024-03-11

**Authors:** Nadia Rodríguez-Medina, Jonathan Rodríguez-Santiago, Alejandro Alvarado-Delgado, Alan Sagal-Prado, Jesús Silva-Sánchez, Miguel A. De la Cruz, Miguel Angel Ares, Margarita Sánchez-Arias, Rayo Morfín-Otero, Rigoberto Hernández-Castro, Patricia Cornejo-Juárez, Emmanuel Jiménez-Villanueva, Domingo Sánchez-Francia, Ulises Garza-Ramos

**Affiliations:** 1 Centro de Investigación Sobre Enfermedades Infecciosas (CISEI), Laboratorio de Resistencia Bacteriana, Instituto Nacional de Salud Pública (INSP), Av. Universidad # 655, Col. Santa María Ahuacatitlán, C.P. 62100 Cuernavaca, Morelos Mexico; 2 Facultad de Medicina, Benemérita Universidad Autónoma de Puebla, Puebla, Mexico; 3 Unidad de Investigación Médica en Enfermedades Infecciosas y Parasitarias, Hospital de Pediatría, Centro Médico Nacional Siglo XXI, Instituto Mexicano del Seguro Social, Mexico City, Mexico; 4 Hospital Civil de Guadalajara “Fray Antonio Alcalde”, Instituto de Patología Infecciosa y Experimental, Universidad de Guadalajara, Guadalajara, Jalisco Mexico; 5 Departamento de Agentes Patógenos, Hospital General “Dr. Manuel Gea González”, Mexico City, Mexico; 6 Instituto Nacional de Cancerología, Mexico City, Mexico; 7 Hospital Regional de Alta Especialidad Ciudad Salud, Tapachula, Chiapas Mexico; 8 Hospital del Niño y el Adolescente Morelense, Zapata, Morelos Mexico

**Keywords:** Virulence, Capsule, Plasmids, Hypermucoviscosity, Colistin-resistance, Bacterial genetics, Pathogens, Comparative genomics, Genetics, Microbiology

## Abstract

Here, we conducted a comprehensive analysis of 356 *Klebsiella pneumoniae* species complex (KpSC) isolates that were classified as classical (cl), presumptive hypervirulent (p-hv) and hypermucoviscous-like (hmv-like). Overall, *K. pneumoniae* (82.3%),* K. variicola *(2.5%) and* K. quasipneumoniae *(2.5%) were identified. These isolates comprised 321 cl-KpSC, 7 p-hv-KpSC and 18 hmv-like-KpSC. A large proportion of cl-KpSC isolates were extended-spectrum-β-lactamases (ESBLs)-producers (64.4%) and 3.4% of isolates were colistin-resistant carrying carbapenemase and ESBL genes. All p-hv-KpSC showed an antibiotic susceptible phenotype and hmv-like isolates were found to be ESBL-producers (8/18). Assays for capsule production and capsule-dependent virulence phenotypes and whole-genome sequencing (WGS) were performed in a subset of isolates. Capsule amount differed in all p-hv strains and hmv-like produced higher capsule amounts than cl strains; these variations had important implications in phagocytosis and virulence. Murine sepsis model showed that most cl strains were nonlethal and the hmv-like caused 100% mortality with 3 × 10^8^ CFUs. Unexpectedly, 3/7 (42.9%) of p-hv strains required 10^8^ CFUs to cause 100% mortality (atypical hypervirulent), and 4/7 (57.1%) strains were considered truly hypervirulent (hv). Genomic analyses confirmed the diverse population, including isolates belonging to hv clonal groups (CG) CG23, CG86, CG380 and CG25 (this corresponded to the ST3999 a novel hv clone) and MDR clones such as CG258 and CG147 (ST392) among others. We noted that the hmv-like and hv-ST3999 isolates showed a close phylogenetic relationship with cl-MDR *K*. *pneumoniae*. The information collected here is important to understand the evolution of clinically important phenotypes such as hypervirulent and ESBL-producing-hypermucoviscous-like amongst the KpSC in Mexican healthcare settings. Likewise, this study shows that *mgrB* inactivation is the main mechanism of colistin resistance in *K*. *pneumoniae* isolates from Mexico.

## Introduction

The *Klebsiella pneumoniae* species complex (KpSC) encompass seven closely related species, of which *Klebsiella pneumoniae*, *Klebsiella variicola* and *Klebsiella quasipneumoniae* are the most frequently reported in healthcare-associated infections^1,2^. Unlike other members of the KpSC, *K. pneumoniae* is recognized by the World Health Organization as a public health threat because it is associated with high rates of antimicrobial resistance (AMR) genes^3^.

Most of the hospital acquired infections are caused by the opportunistic pathogen commonly referred as classical (cl) while the pathogen type cause severe infections, sometimes in multiple sites in healthy individuals, this variant is referred as hypervirulent (hv)^1^. Both represent two well-recognized phenotypes in *K. pneumoniae* and they can also be distinguished in other members of the KpSC.

Features associated with cl phenotype are the presence of multiple AMR genes such as extended-spectrum β-lactamases (ESBL), carbapenemases encoding genes and colistin-resistance mechanisms and in patients an immunocompromised condition that increases the risk for cl-KpSC infections^4^. In contrast, strains with hv phenotype possess fewer AMR genes, but several plasmid-mediated virulence genes promotes the highly virulent phenotype. These include the siderophores aerobactin (*iuc*/*iutA*), yersiniabactin (*ybt*/*irp*) and salmochelin (*iro*), the genotoxin colibactin (*clb*), microcin E492 and *rmpADC*/*rmpA2* genes; the latter are key drivers of increased capsule production and hypermucoviscosity^4^. The siderophores and mucoid regulators have been attributed as main factors to the hypervirulent phenotype^5,6^. We agree with Kochan et al. that the criteria to distinguish between hv-Kpn and cl-Kpn strains are challenging and the definitions of these groups are controversial^6^. The siderophores aerobactin, salmochelin and the regulator operon *rmpADC* are main virulence factors present in the majority of hypervirulent strains. Furthermore, mouse infection models are recommended to accurately distinguish between hv-Kpn and cl-Kpn^6^.

The increasing reports of strains from the KpSC showing hypermucoviscous (hmv) phenotype not related to *rmpADC* supports the notion of a third phenotype^2,7–13^; hereafter referred to as hmv-like. This population of strains exhibit copious amounts of capsule, but the genetic basis is still unknown and deserves to be studied in detail as it might have clinical implications in *Klebsiella* infections. Moreover, the genomic features and phylogenetic position within the KpSC has been little explored.

Here, we conducted a retrospective multicenter study of isolates belonging to the KpSC. We provided a comprehensive analysis in order to report the phenotypes diversity occurring in Mexican hospitals by assessing its antimicrobial susceptibility, pathogenicity, molecular epidemiology and genomic traits.

## Results

### Distribution of clinical pathogens from the KpSC

**Figure 1:**
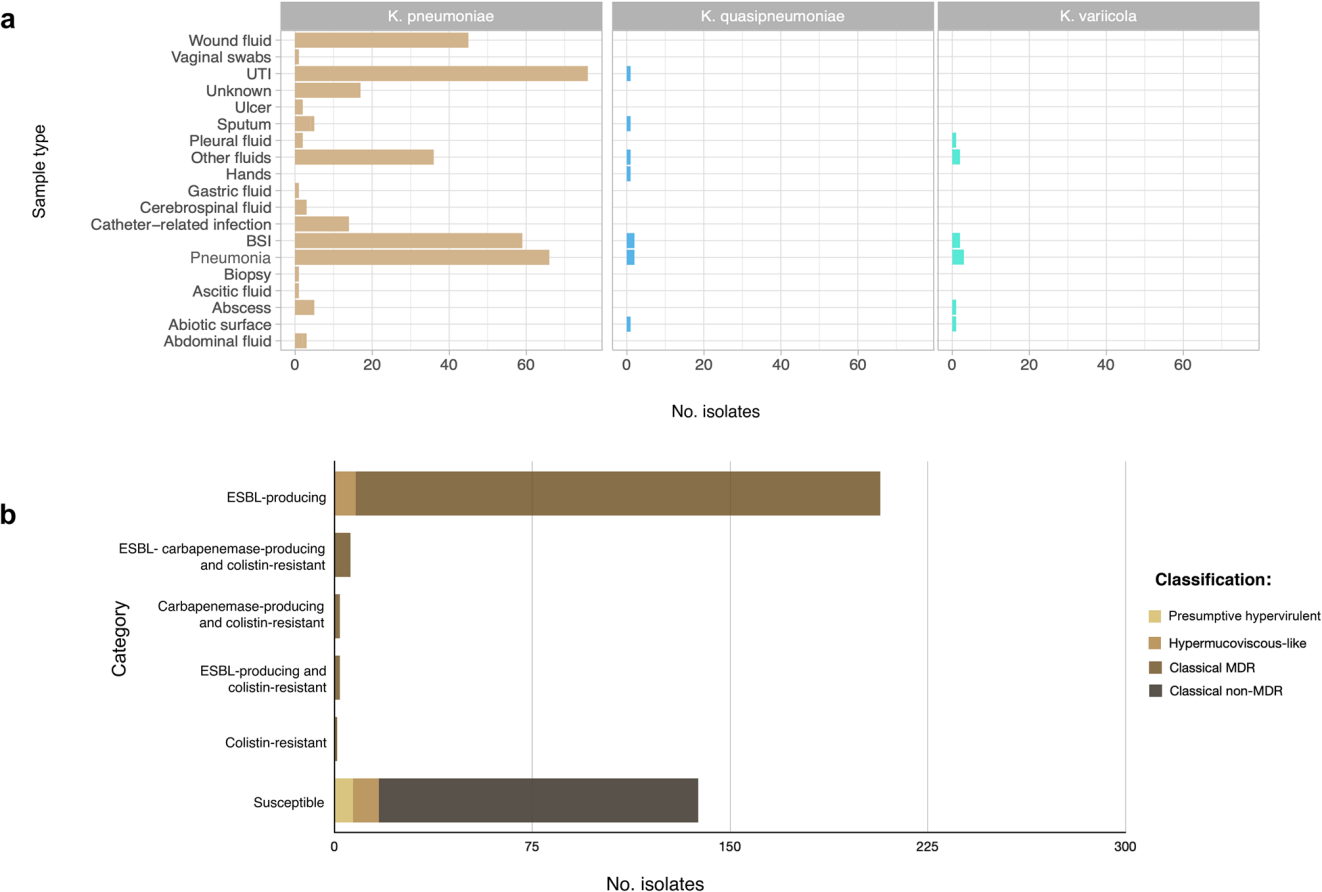
(**a**) Species distribution and sample types of 356 KpSC isolates. (**b**) Occurrence of presumptive hypervirulent, hypermucoviscous-like and classical phenotypes amongst 356 isolates of KpSC.

This study included 356 K*. pneumoniae* isolates collected from February 2014 to December 2017 from eleven different healthcare settings and eight cities in Mexico (Supplementary Fig. 1). Multiplex PCR confirmed that the majority of the 356 isolates were classified as *K. pneumoniae* (95%, n = 338/356); the rest were identified as *K. quasipneumoniae* (2.5%, n = 9/356) and *K. variicola* (2.5%, n = 9/356) (Table 1). The *K. pneumoniae* isolates were obtained mainly from patients with episodes of urinary tract infections (UTI), blood stream infections (BSI), pneumonia and wound infection (Fig. 1a). Most isolates were collected from internal medicine and surgery wards (Supplementary Fig. 1).

**Table 1:**
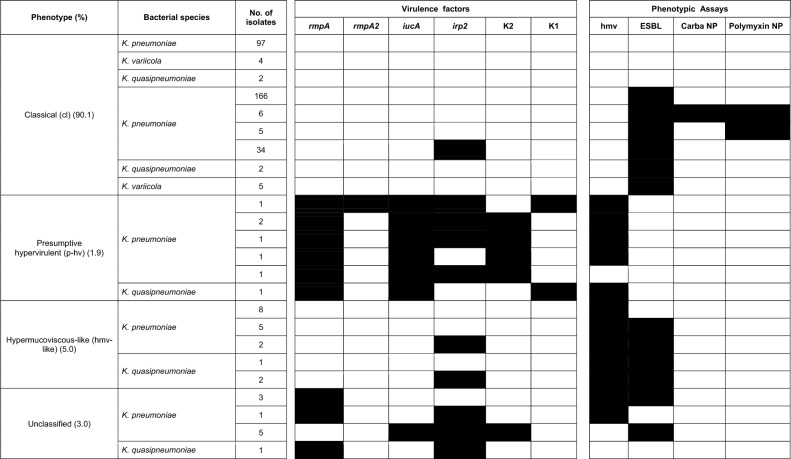
Prevalence and characteristics of classical, presumptive hypervirulent and hypermucoviscous-like isolates from the KpSC.

The virulence factors were PCR-amplified using specific primers as detailed in Supplementary Table 1. The hmv phenotype was initially tested using the qualitative string test, and ESBL confirmation was performed using Kirby Bauer, carbapenemase producing by Carba NP and colistin-resistance by Polymyxin NP methods.

### Phenotypes diversity amongst KpSC isolates

The KpSC isolates were classified as cl, presumptive hv (p-hv) or hmv-like according to the virulence markers and string test (Table 1); also the antimicrobial susceptibility was determined in the total number of isolates listed in Table 1.

The cl phenotype was the most prevalent (90.1%, n = 321/356) in which 218 isolates of 321 consisted of cl-MDR strains and 103 isolates of 321 were classical non-MDR strains. The cl-MDR phenotype comprised ESBL-producing isolates (n = 207/218) and ESBL and/or carbapenemase-producing colistin resistant isolates (n = 11/218) (Fig. 1b). The genotype *bla*_CTX-M-15,_ *bla*_NDM-1_, *bla*_KPC-2_, and *bla*_OXA-232_ explained the ESBL- and carbapenemase-producer phenotype; disruptions and mutations in the regulatory gene *mgrB* and missense mutations in *pmrA* and *phoQ* genes explained the colistin resistance phenotype (Supplementary Table 1). Five clonal-related (clone A) colistin-resistant *K. pneumoniae* isolates co-producing the carbapenemase NDM-1 and the ESBL CTX-M-15 were identified. Interesingly, the insertion sequence (IS)-mediated disruption of the *mgrB* gene identified in isolate 13861 (ISKpn26) is different from those identified in the isolates 7040 and 14669 (IS1X2); this indicates the acquisition of colistin resistance by IS-mediated disruption of the *mgrB* gene was an independent event in isolate 13861 (Supplementary Table 1).

The p-hv and hmv-like isolates were identified in 1.9% (n = 7/356) and 5.0% (n = 18/356) of *Klebsiella* infections, respectively (Fig. 1b). The p-hv phenotype was noted in six *K*. *pneumoniae* and one *K*. *quasipneumoniae* subsp. *similipneumoniae* isolates (Table 1). Clinical characteristics revealed that p-hv strains were obtained from community- and hospital-acquired infections (Supplementary Table 2). The hmv-like phenotype was observed in *K*. *pneumoniae* (77.7%, n = 15/18) and *K*. *quasipneumoniae* (22.3%, n = 3/18) and 10 of 18 were ESBL-producers (Table 1 and Fig. 1b).

### Assessment of capsule production, phagocytosis, serum killing and biofilm

**Figure 2:**
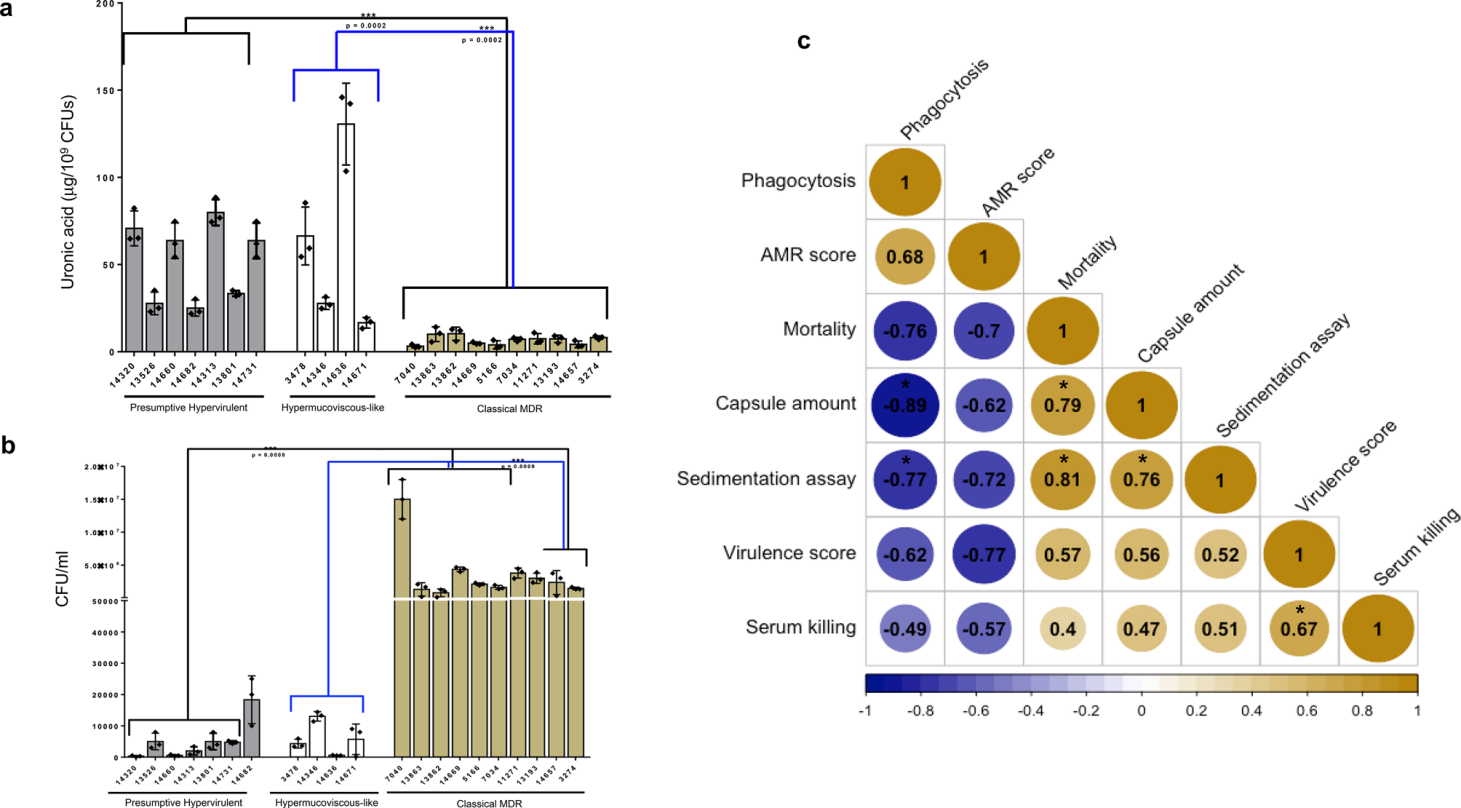
Assays for evaluating capsule production and phagocytosis**. **(**a**) Quantification of uronic acid and (**b**) Phagocytosis killing assays for presumptive hypervirulent, hypermucoviscous-like and classical MDR KpSC isolates. * Indicate *p* < 0.0002. Bars represent the mean levels ± 95%. (**c**) Correlation matrix of virulence traits. Numbers indicate Spearman’s correlation and stars identify significant correlations (*p* < 0.05). A − 1 value represent a strong negative correlation and a + 1 value a strong positive correlation.

Quantification of capsule by means of uronic acid measurement revealed that all cl isolates produced less capsule than p-hv and hmv-like isolates (*p* < 0.0002) and in some instances the hmv-like isolates produced as much capsule or more as the p-hv ones (Fig. 2a). Although the p-hv isolates showed greater capsule production than cl ones, we noted variable capsule production profiles amongst this group; 3 of 7 p-hv isolates produced less capsule (Fig. 2a), pointing out that capsule regulation via *rmpA* might be impacted. Hence, we looked for mutations in *rmpA* genes that could explain the lower production of capsule in 13526, 14682 and 13801 isolates. We observed that 13526 presented two insertions in positions 285 (poly-(G) tract) and 461 which leads to a frameshift mutation, but for 14682 and 13801 the *rmpA* sequence was the same as the wild type (Supplementary Fig. 2).

Phagocytosis assay showed a negative correlation with capsule production, so those isolates with higher capsule amounts were less phagocyted (Fig. 2b and Fig. 2c). In contrast, serum killing was independent of capsule production, but capsule-type dependent. All p-hv-KL1 isolates were serum-resistant, while one hmv-like-KL54 isolate and one cl-KL2 isolate resisted complement killing (Supplementary Table 3). Serum killing positive correlated with virulence score (Fig. 2c).

Biofilm formation indicate that all isolates had an index < 1 except the *K. pneumoniae* 5166 isolate which showed 1.0 (Supplementary Table 3). Biofilm formation at least in the isolates included in this study was not related with cl, hmv-like and hv phenotypes. Therefore, this trait cannot be used as a criterion to differentiate these phenotypes.

### Evaluation of virulence

**Figure 3:**
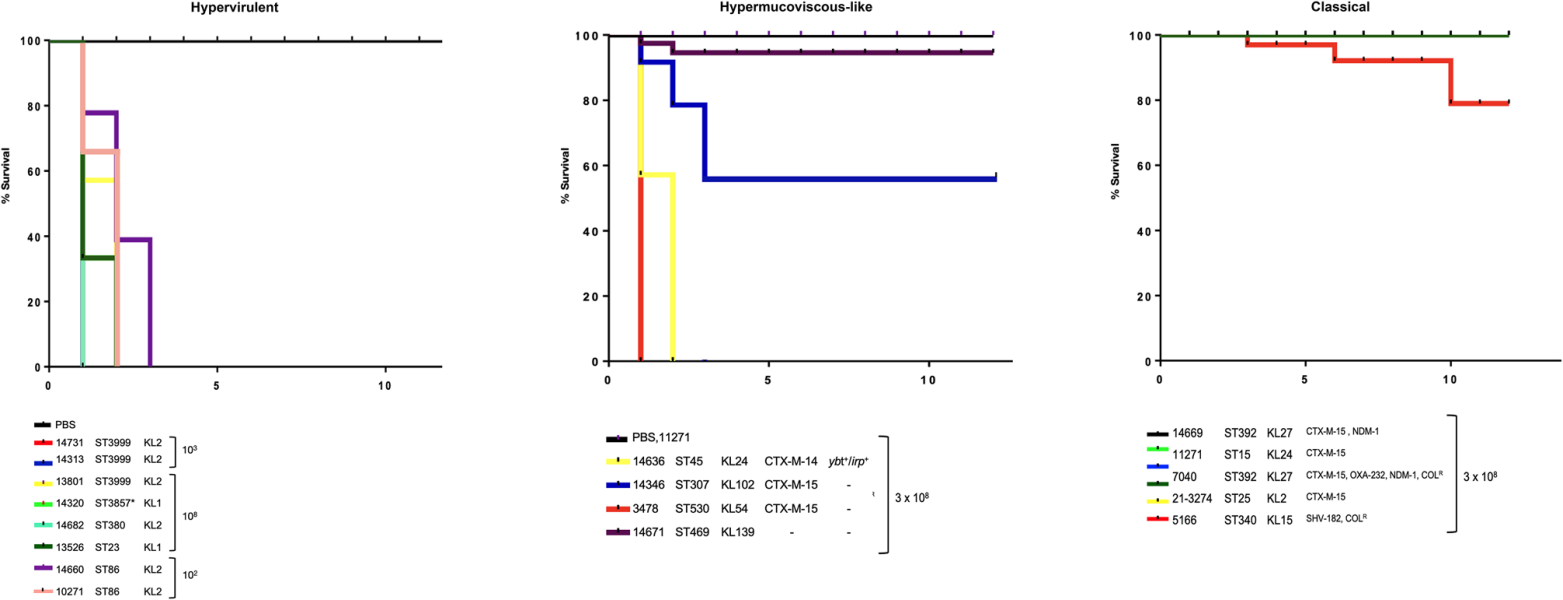
Kaplan–Meier plots showing the percent survival of BALB/c mice over twelve days post-infection with hypervirulent and atypical hypervirulent (10^2^, 10^3^ and 10^8^ CFUs), hypermucoviscous-like (3 × 10^8^ CFUs), and classical (3 × 10^8^ CFUs) strains. The characteristics of each strain such as ST, capsular type and ARGs are included. The panel of classical isolates included two colistin resistant isolates (COL^R^) due to *mgrB* disruption (14669) and mutations in *pmrB* gene (5166). Mice challenged with classical MDR strain 11271 and the hypervirulent 10271 ST86-KL2 were used as controls^18^. *This isolate correspond to hv *K. quasipneumoniae* subsp. *similipneumoniae*.

To accurately determine hypervirulent strains and differences in virulence levels within the p-hv, hmv-like and cl phenotypes a murine sepsis model was implemented. We observed variable virulence profiles in mice infected with p-hv strains. At first, we inoculated mice with 10^2^ and 10^3^ CFUs of all p-hv KpSC isolates, but only the isolates 14731, 14313 and 14660 showed 100% mortality; hence, they were defined as hypervirulent (Fig. 3). However, the isolates 13801, 14682, 13526 and 14320 showed no mortality at lower doses and therefore a broader range of bacterial loads (10^4^ to 10^8^ CFUs) was tested. We found that these isolates exhibited 100% mortality at 10^8^ CFUs (Fig. 3), thus they were defined as atypical hypervirulent (Supplementary Table 3). There is one exception that applies for *K. quasipneumoniae* 14320 which is not expected to have a similar virulence level as hv-Kpn.

We next investigated the virulence levels for hmv-like and cl-MDR *K. pneumoniae* strains. We found that only the hmv-like strains 14636 and 3478 showed 100% mortality at 3 × 10^8^ CFUs; these strains displayed the highest production of uronic acid (Fig. 2a) while the 14346 strain was moderately virulent (Fig. 3). Finally, no mortality was observed in mice challenged with 3 × 10^8^ CFUs of cl strains except for the colistin-resistant 5166 isolate (Fig. 3 and Supplementary Table 3).

### Molecular epidemiology of KpSC isolates

To place our isolates in a global context, we study the molecular epidemiology defined by 7-locus MLST and performed a phylogenetic analysis including reference strains as detailed in Material and Methods section.

**Figure 4:**
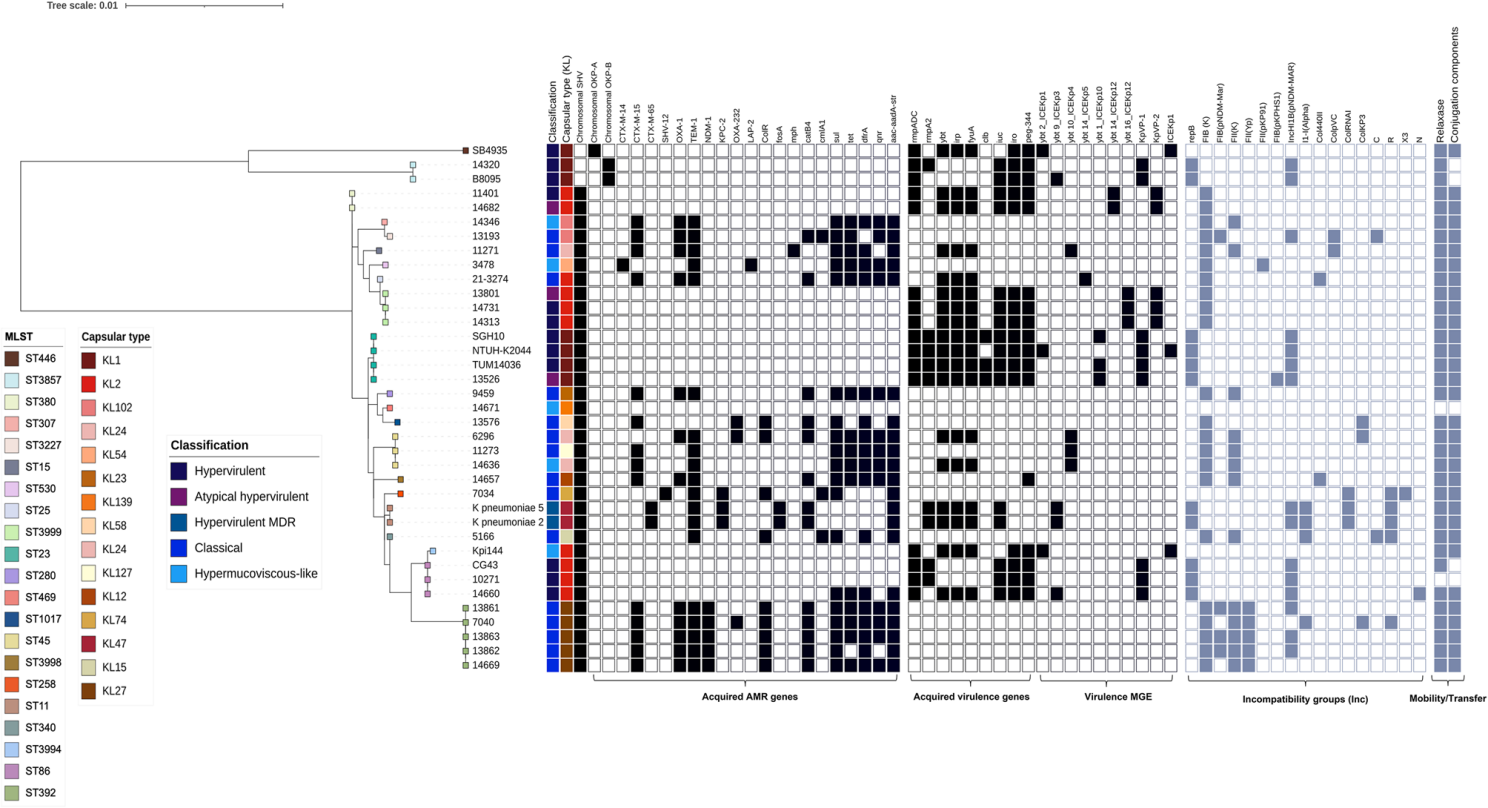
Molecular epidemiology of KpSC isolates. The phylogenetic tree was constructed with the concatenated sequence of the seven MLST genes. Repertoire of virulence and AMR genes, plasmid types and mobility/transfer components are shown as a presence and absence matrix.

Our collection of KpSC isolates was genetically diverse, comprising different sequence types (ST) some of them from high-risk clones (Fig. 4). hv-KpSC isolates were comprised by ST23-KL1 (CG23), ST86-KL2 (CG86) and ST380-KL2 (CG380), which are well-known virulent clones. Curiously, we noted that three isolates were ST3999; this ST was a single locus variant (SLV) of the ST25, so both sequence types belong to CG25. To the best of our knowledge, this is the first time that ST3999 is associated with the hypervirulent genotype.

The hv *K*. *quasipneumoniae* subsp. *similipneumoniae* 14320 clustered with a previously reported hypervirulent *K*. *quasipneumoniae* isolate from India (Fig. 4). Both isolates have the KL1 capsular type.

The hmv-like *K. pneumoniae* isolates showed diverse STs and capsular types, and in some instances, cl and hmv-like isolates shared the same ST, e.g. 11273 and 14636 both with ST45 (Fig. 4). cl-*K. pneumoniae* isolates were distributed in ten STs, of which we identified ST258-KPC-2-producing and ST392-NDM-1-producing and colistin-resistant isolates (Fig. 4 and Supplementary Table 1).

### Virulome, resistome and plasmid repertoire

We investigated the repertoire of virulence and AMR genes, and found that virulence genes tend to accumulate in hv and atypical hv isolates. In contrast, acquired AMR genes were present in cl and hmv-like isolates (Fig. 4). The siderophore yersiniabactin was distributed not only in hv strains but also in cl and hmv-like *K*. *pneumoniae*. Convergence of hmv-like phenotype and ESBL production (CTX-M-14) was observed in the 14636 isolate that additionally carried the *ybt* locus (yersiniabactin) (Fig. 4). We noted that the ST23 isolates: SHG10, TUM14036 and 13526 were clustered together, and all possessed the colibactin toxin.

We used multiple approaches to assess the plasmid content in our genome collection. First, plasmid replicons were detected using the PlasmidFinder database. Two replicons were identified in the hv-KpSC group (encompassing ST23-KL1, ST86-KL2 and *K. quasipneumoniae* ST3857-KL1), repB and IncHI1B(pNDM-MAR) and one unique replicon was associated with hv-Kpn ST3999-KL2 and ST380-KL2, IncFIB_K_ (Fig. 4). cl and hmv-like *K*. *pneumoniae* isolates displayed plasmid replicons from the F and Col families as detailed in Fig. 4**.** Of note, IncFIB_K_ plasmids were associated with CTX-M-14 and CTX-M-15 and IncFII_Yp_, IncFII_K_, IncFIB_K_ plasmids were linked with NDM-1 and KPC-2 carbapenemases. As expected, cl isolates possessed more plasmid replicons whereas the other *K. pneumoniae* variants did not.

Second, MOB proteins (those implicated in plasmid mobility) and conjugation proteins (those involved in matting pair formation) were investigated since both have a role in AMR and virulence transmission. All but two genomes contained proteins implicated in conjugation process (Fig. 4 and Supplementary Table 4) incluing the hv CG23, CG86, CG380 and CG25 and all but one genome contained one of the eight relaxase MOB families (Fig. 4 and Supplementary Table 4). It is well known that hypervirulence-plasmids are non-conjugative, but virulence genes are usually located within an ICE having the ability to mobilize and/or conjugate. We observed that there were few cases where no conjugation proteins were detected, but for which a relaxase was found (Fig. 4).

Third, the kleborate typing tool reports associated-virulence MGE variants which can provide undersating about dissemination. The virulence plasmid type KpVP-2 was a dominant type amongst the ST380- and ST3999-hv *K. pneumoniae*, but we also identified other MGEs such as KpVP-1 in *K*.*pneumoniae* 14660, 13526 and *K*. *quasipneumoniae* 14320. No integrative elements, for example the well-described ICE*Kp*-1 were found in the genomes from this study (Fig. 4).

### Phylogenomic analysis

**Figure 5:**
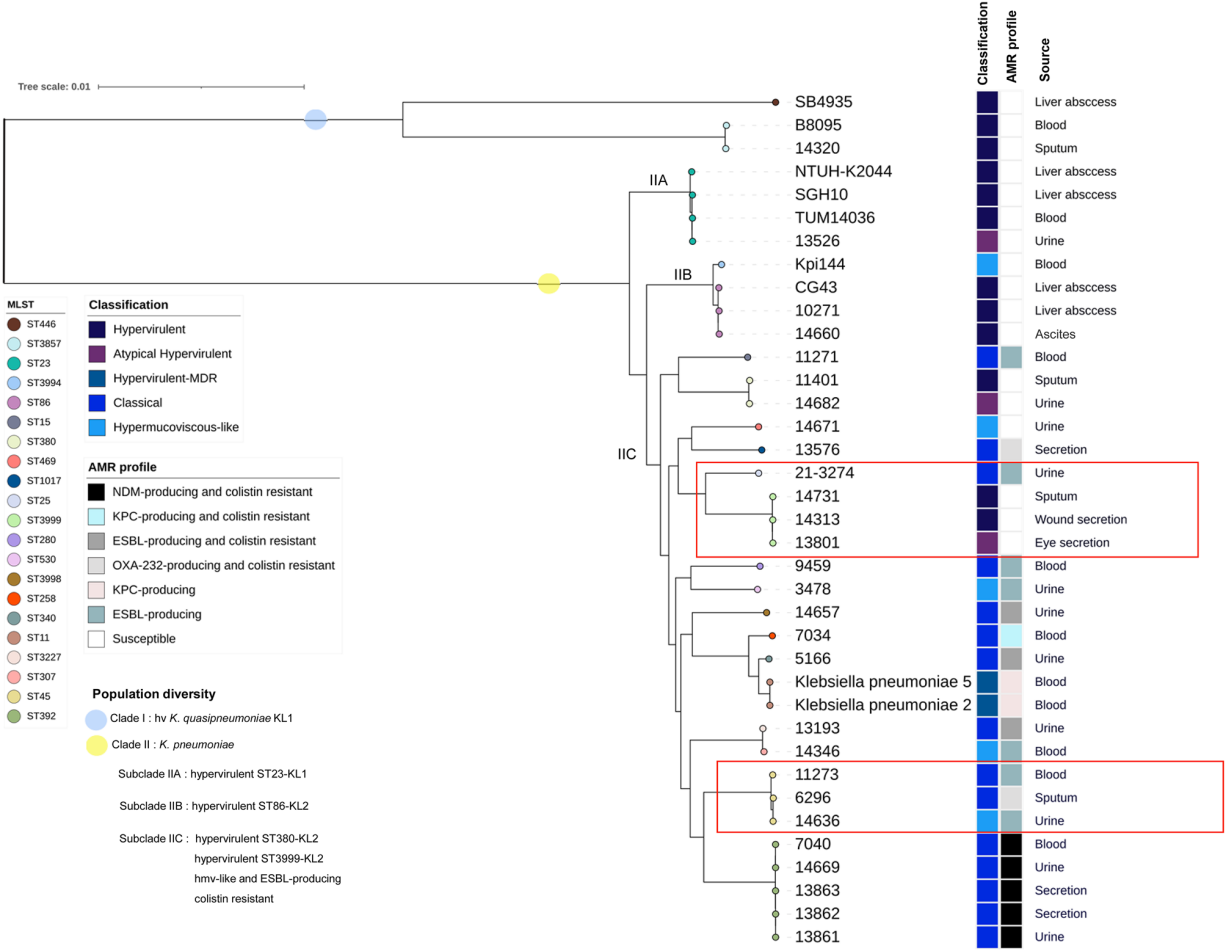
Phylogenomic diversity of KpSC isolates. Whole-genome SNP-based tree inferred with maximum likelihood approach. The classification, AMR profile and source of each isolate is denoted in the tree. An antibiotic susceptible profile is denoted as white boxes. Clades in which hv or hmv-like isolates clustered with ESBL- and/or carbapenemase-producing *K. pneumoniae* are highlight in red.

The phylogenomic diversity was analyzed through a maximum likelihood whole-genome SNP tree. Our analysis clustered the 37 genomes into two major clades represented by hv *K*. *quasipneumoniae* KL1 isolates (clade I) and *K*. *pneumoniae* isolates (clade II) (Fig. 5). The clade II was split into three main subclades: subclade IIA was primarily composed of hv isolates from the ST23 and capsular type KL1, subclade IIB included hv isolates from the ST86 and capsular type KL2 and subclade IIC comprised hv, hmv-like and cl isolates (Fig. 5). We found that hv and two atypical hv isolates within subclade IIC were closely related with ESBL-producing and colistin-resistant isolates. For instance, hv-ST3999 and hv-ST380 were closely related with CTX-M-15 producing isolates, whereas the reference hv-ST11 isolates were closely related with colistin resistant and carbapenemase-producing isolates from our study. Similarly, hmv-like isolates formed a clade with ESBL- and colistin-resistant and carbapenemase-producing isolates (Fig. 5).

## Discussion

Members of the KpSC share genetic information allowing them to evolve, so features that were only seen in *K. pneumoniae* are now seen in *K*. *variicola* or *K. quasipneumoniae*. Several reports of *K*. *variicola* and *K*. *quasipneumoniae* that acquired AMR genes and gained the ability to cause invasive infections supports this fact^2,14,15^.

In this study, the distribution and characteristics of cl, hv and hmv-like isolates of the wider KpSC occurring in Mexican hospitals was explored in a comprehensive approach.

In the first instance, the ESBL-producing isolates are still the most frequently identified amongst the KpSC (58.1%), and they represent the dominant phenotype in our collection. The acquisition of mechanisms for colistin resistance amongst ESBL- and carbapenemase-producing KpSC isolates implies the potential emergence of microorganisms resistant to all antimicrobials available (pan-drug resistant). Rodríguez-Santiago et al. reported that 95.3% of the carbapenemase-producing and colistin-resistant Enterobacterales in the Americas correspond to *K*. *pneumoniae*, and the KPC-type carbapenemase was the most frequently identified^16^. In this study, the primary mechanism of colistin-resistant isolates was modifications in the *mgrB* gene, and the main carbapenemase was NDM-1. Unlike the panorama in different regions of Latin America, a recent study carried out in Mexico showed that the NDM-type carbapenemases were more frequent than KPC-type^17^. Thus, we consider that Mexico is New-Delhi metallo-β-lactamase territory.

The colistin resistance in clinically relevant strains of *K. pneumoniae* imply additional challenges to health care and are scarcely described in Mexico. Currently, work is being carried out to study the genetic and phenotypic implications of colistin-resistance mechanisms in hv and cl-MDR *K*. *pneumoniae* isolates from Mexico.

A few reports in Latin America of hv *K*. *pneumoniae* K2 strains exist^18–20^, but no reports of hv *K*. *pneumoniae* or *K. quasipneumoniae* with capsular type K1 have been described in Mexico until this report. The lineage belonging to ST23-K1 constitutes the majority of hv-Kpn; however, the hv strains in our sample collection were diverse as we observed multiple STs and variable capsule-dependent virulence phenotypes (Fig. 2 and Fig. 3). These strains were recovered from different hospitals settings and geographic locations, and in one hospital circulated four different hv-associated STs (Supplementary Fig. 1 and Supplementary Table 2). We suspect that the introduction of hypervirulent isolates was through independent events of clonal dissemination.

We found that capsule amount and phagocytosis assays were strongly associated with virulence (Fig. 2c). Thus, the hv and hmv-like strains that produced more capsule were less phagocyted and displayed higher mortality rates (Figs. 2 and 3).

Amongst the presumptive hv-Kpn isolates, we observed three instances of atypical hv isolates (13526, 14682 and 13801) which required a higher bacterial load (10^8^) to cause mortality (Fig. 3). This phenomenon most likely reflects the linkage with low capsule production and high rate of internalization by macrophages. We suspected that frameshift mutations in *rmpA* could be altering the capsule production. The isolate 13526 (ST23-KL1) presented indels that occurred at the same positions as the report by Yu et al.^13,^ but we could not explain the impairment in capsule production in 14682 and 13801; however, mutations occurring in the promoter region of *rmpA* could be responsible for capsule production defects. It is notable that the presence of hypervirulence-associated genes did not necessarily lead to hypervirulence phenotype.

We observed genotypes that were not categorizable according to our classification framework. For instance, hyperpemucoviscous isolates positive to *rmpA* but no other hv-associated genes or isolates positive to *iucA*, *irp2* and capsular type K2 but negative to *rmpA* and hypermucoviscosity (Table 1); these isolates cannot be considered as truly hypervirulent as there is lack of evidence about the minimal virulence repertoire needed for hypervirulence phenotype expression.

Plasmids play an important role in the emergence of new clones or phenotypes such as is the case of the hv *K. quasipneumoniae* 14320 isolate and the novel hv ST3999. Both emerge due to the acquisition of the virulence plasmids KpVP-1 and KpVP-2, respectively; these plasmid variants are non-conjugative, but recent studies indicate that they can be mobilized with the help of other plasmids^21^. Another scenario may have been the acquisition of a variant plasmid that was capable of self-transfer in some point of the evolutionary history of each strain. Further experiments and complete genome sequencing are required for evaluating the conjugation or mobilization ability of both virulence plasmids.

Interestingly, the CG25 was identified as the most diverse hv clone in terms of the acquired AMR genes and the higher recombination rates in comparison to other hv clones^22^, so it make sense that STs within this clonal group, such as the ST3999, are more apt to acquire plasmids carrying virulence or AMR genes. This is also consistent with the observation in our phylogenetic analysis in which ST3999 isolates shared a close genetic relationship with ST25/CTX-M-15-producing isolates (Fig. 5). CG25 is of great alert as convergent strains may arise from this clonal group^22,23^.

Phylogenetic analysis also demonstrates that cl-MDR *K*. *pneumoniae* shared a close genetic relationship with hmv-like isolates which formed a clade with colistin resistant and ESBL-producing isolates (Fig. 5). Convergence events of multidrug resistance and virulence has been reported in numerous studies^1,6,24^ and genomic evidence has shown that MDR clones are more likely to become virulent or highly virulent^22^.

A good example is the outbreak cause by the hypervirulent and KPC-2-producing ST11 *K. pneumoniae* in China^25^. These isolates were included in our phylogenetic analysis and were located in the subclade IIC, same subclade that comprised cl-MDR, hmv-like and hv *K*. *pneumoniae*. It is plausible that classical strains in clinical scenarios gain virulence determinants or gain hypermucoviscosity as our hmv-like isolates. Recently, Kochan et al. studied *K. pneumoniae* convergent isolates and identified that these isolates with both multidrug-resistance and hypervirulence had unexpectedly low virulence^6^. In this study, no convergent isolates were identified; however, the atypical hv *K. pneumoniae* 13526 (KL1 and ST23), 14682 (KL2 and ST86) and 13801 (KL2 and ST3999) had an unexpectedly low virulence compared to the hv 14660 and 10271 (hv control strain)^18^ (Fig. 3).

The genetic basis of hmv-like is unknown, but some authors have suggested that unknown plasmid-mediated and chromosomal determinants are responsible for this sort of phenotype implying that independent mechanisms are involved in non-*rmpADC* related hypermucoviscosity^7,8^. This phenotype has a role in virulence by preventing adherence and internalization of *K. pneumoniae* by macrophages^26^. In our study, the mice infected with those isolates with the highest production of hypermucoviscosity had increased mortality as compared to cl-MDR-Kpn (Fig. 3), these data are similar to those reported by Rodríguez-Medina et al.^8^, when infecting mice with hmv-like *K. variicola*. These findings illustrate that hmv-like KpSC represent a population to be aware of, since hypermucoviscosity is contributing to virulence and AMR and other virulence-associated genes are converging in this genetic background.

Taken these results together, we show that phenotypic heterogeneity occurs within each phenotype in terms of capsule production, virulence and antimicrobial resistance. The molecular mechanisms or environmental signals that influence these variations are not covered in this study but are the focus for future research.

## Conclusions

Members of the KpSC possess a critical challenge to healthcare due to its ability to acquire virulence genes and resistance to multiple antibiotic families. Our results illustrates the pattern of phenotypes and species diversity occurring in a collection of isolates from the KpSC. The ESBL-producing isolates are still the most frequently identified bacteria amongst the KpSC.

The circulation of hv strains was due to independent events since different genetic backgrounds and STs were found. Furthermore, our study indicates that the presence of hv-associated genes was not sufficient to make the assumption of hypervirulence and it was necessary to integrate infection models. On the other hand, strains displaying the hmv-like phenotype are potential threats that need further investigation as there is evidence that high production of hypermucoviscosity contributes to virulence.

Although the occurrence of hv, hmv-like and colistin-resistant KpSC isolates was low, the information collected here is important to understand how the frequency and evolution of these strains may changed with time. This study serves as baseline information to monitor current phenotypes and variations within themselves in health care settings.

## Material and methods

### Sample collection, bacterial species identification and fingerprinting analysis

A total of 403 K*. pneumoniae* isolates were collected from February 2014 to December 2017. We received from healthcare settings presumptive *K*. *pneumoniae* isolates that were identified by biochemical tests or automated microbiological systems. Susbsequently, the proper differentiation of the main members of the KpSC was done by multiplex PCR^27^. Forty-seven isolates were eliminated from the study because they were duplicated or could not be assigned as *K. pneumoniae, K. quasipneumonia*e or *K. variicola* or belonged to a bacterial species outside of the KpSC. Thus, the total isolates was 356. The genetic relatedness of selected isolates was examined by pulsed-field gel electrophoresis (PFGE) and the results were analyzed using GelCompar II software (Applied Maths, Kortrijk, Belgium).

### PCR-amplification of virulence genes screening and antimicrobial susceptibility

All strains were screened for *rmpA, rmpA2*, *iucA* and *irp2* genes and for capsular types K1 and K2 (Supplementary Table 5). All isolates were tested for ESBL-, carbapenemase-producing and colistin resistance phenotype by using Kirby-Bauer, CarbaNP and Rapid Polymyxin NP assays. The isolates positive in the Rapid Polymyxin NP assay were confirmed by minimal inhibitory concentration (MIC) using the broth microdilution procedure, accordingly to the EUCAST breakpoints^28^.

### Classification of classical, presumptive hypervirulent and hypermucoviscous-like phenotypes

We classified KpSC isolates as classical (cl), hypermucoviscous-like (hmv-like) and presumptive hypervirulent (p-hv), considering recently reported criteria^6,29,30^. p-hv strains were defined as any strain positive for *rmpA* and *iucA* genes; and/or the presence of *rmpA2*, *irp2*, K1, K2. hmv-like strains corresponded to those positive for string test but negative for *rmpA* or *rmpA2* genes*.* Isolates lacking p-hv or hmv-like characteristics were categorized as cl strains. Those isolates that present a limited virulence repertoire, without enough evidence to be associated with a truly hypervirulent phenotype, remain as unclassified. The murine sepsis model was also implemented for confirming the identification of hypervirulent strains, and those with lower virulence in terms of CFUs load were considered atypical hypervirulent.

### Assays for capsule production and capsule-dependent virulence phenotypes

We implemented the methods proposed by Walker and Miller^31^ for evaluating virulence and capsule production. For these assays we select all presumptive p-hv isolates (n = 7), four hmv-like isolates (Supplementary Table 2) and ten classical isolates selected on basis of the isolation source, clonality and antimicrobial susceptibility profile. Mutations in *rmpA* gene were search as follows: we extracted the sequences of the *rmpA* genes of the seven hv isolates and aligned them against the *rmpA* sequence from the reference strains NTUH-K2044 and SGH10 using muscle aligner. The sequence was inspected using iTOL.

### Quantification of uronic acid and sedimentation assays

Capsular polysaccharides were extracted and quantified using a colorimetric assay for uronic acid as previously described^8^. In addition to the qualitative string test, the sedimentation assay was assessed according to Bachman et al.^32^. An overnight culture was pelleted by centrifugation at 8000×*g* and resuspended in phosphate-buffered saline (PBS) to an OD_600_ of ∼1. The suspensions were centrifuged for 5 min at 1000×*g*, and the OD_600_ of the supernatants was measured. Final readings were normalized to the OD_600_ of the wild-type culture before centrifugation. The results are presented as the mean and standard deviation of the data of three experiments.

### Phagocytosis resistance and serum killing assays

The phagocytosis assay was performed using THP-1 (ATCC TIB-202) human monocytes (differentiated to macrophages with 200 nM of phorbol 12-myristate 13-acetate for 24 h) were seeded (6 × 10^5^) into 24-well tissue culture plates. Bacteria were grown in 5 mL of LB, LBT, and LBC to the exponential phase (2 h, OD_600nm_ = 0.8). THP-1 macrophages were infected with a MOI of 100 in a final volume of 1 mL RPMI 1640 tissue culture medium supplemented with 10% heat-inactivated fetal bovine serum. To synchronize the infection, plates were centrifuged at 200 g for 5 min. Plates were incubated at 37 °C under a humidified 5% CO_2_ atmosphere. After 2 h, cells were rinsed thrice with PBS and incubated for an additional 1 h with 1 mL of RPMI 1640 containing 10% FBS and gentamicin (100 μg/mL) to eliminate extracellular bacteria. Cells were then rinsed again thrice with 1 mL of PBS and lysed with 1 mL of 0.1% Triton X-100. After homogenization, tenfold serial dilutions were plated onto LB agar plates to determine total CFU.

For serum killing assays human blood was obtained from healthy volunteers. An inoculum of 25 μl of bacterial suspension (∼10^6^ CFU) prepared from the mid-log phase was mixed with 75 μl of pooled human serum. Viable counts were checked at 0, 1, 2, and 3 h of incubation at 37 °C. Each strain was tested three times, and the mean results were expressed as a percent of surviving inoculum. The response to serum killing in terms of viable counts was scored using six grades classified as serum sensitive (grade 1 or 2), intermediately sensitive (grade 3 or 4), or serum resistant (grade 5 or 6). Inactivated serum at 56 °C was used as negative control^33^.

### Biofilm production

The biofilm assay was performed according to Bandeira et al.^34^. Briefly, the *K. pneumoniae* suspensions at a final concentration of 10^7^ CFU/mL were prepared in 0.9% sodium chloride from overnight cultures in Mueller–Hinton (MH) agar and ten-fold diluted in MH broth. Two-hundred microliters were distributed to each well, MH broth being used as the negative control. The plates were incubated at 37 °C to allow biofilm formation for different time periods. The content was removed and was washed three times with sterile distilled water. The attached bacteria were stained for 15 min with 100 μL of violet crystal at room temperature, washed with distilled water three times to remove excess dye and then dry at room temperature. The violet crystal was dissolved in 100 μL of 95% ethanol and the optical density at 570 nm was read. The *Staphylococcus aureus* ATCC 25,923 was used as positive control and *K. pneumoniae* ATCC 13,883 as negative control. Using the absorbance of planktonic cells and the biofilm, the biofilm index was calculated (A_biofilm_/A_planktonic cells_ = A_570_).

### Mouse infection model

The murine sepsis model was performed in all p-hv, four hmv-like strains and five cl-MDR strains. Groups of five healthy male BALB/c mice were obtained from the animal facility of the National Institute of Public Health at the age of 6–7 weeks and weight between 18 and 24 gr. Briefly, bacteria were grown overnight in LB and were subsequently serially diluted to the required titers in 1X PBS. A 100-μl bacterial suspension was injected intraperitoneally. cl and hmv-like strains were inoculated at 3 × 10^8^ CFUs. Animals were monitored twice daily for twelve days post inoculation. For each, experiment five mice injected with sterile PBS, 11,271 (cl-Kpn) and 10,271 (hv-Kpn) isolates were included as controls.

### Whole genome sequencing (WGS) and bioinformatic analysis

Twenty-six isolates for WGS were selected considering the isolation source, preferably of bloodstream and urinary tract infection and secretion and antibiotic susceptibility profile, and belonged to the cl (n = 15) hv (n = 7), and hmv-like (n = 4) phenotypes (Supplementary Table 2 and Supplementary Table 3). Total genomic DNA was extracted and purified using the DNeasy Kit (Qiagen, Germany). WGS was generated using Illumina (MiSeq) platform. Evaluation of quality assemblies was performed with QUAST (Quality Assessment Tool for Genome Assemblies). Quality-based trimming was performed with the trim galore v.0.4.4 and de novo assembly was done with SPAdes v3.1.1. MLST, virulence genes (*ybt*, *iro*, *iuc*, *rmpA*, *rmpA2*), capsular types (KL), acquired resistance genes and the presence of Mobile Genetic Elements (MGEs) harboring the key virulence genes (ICEKp and virulence plasmids) were typed using Kleborate v0.3.0. The *peg-344* gene was identified using BLASTn taking as query the predicted sequence in the *K. pneumoniae* NTUH-K2044 genome with accession number BAH65947.1. Chromosomal genes associated with colistin resistance in *K. pneumoniae*, including *pmrA*, *pmrB*, *phoP*, *phoQ*, *mgrB*, and *crrB*, were examined for non-synonymous mutations through TBLASTN analysis. Reference amino acid sequences were obtained from the genome of *K. pneumoniae* MGH 78578 (NC_009648.1). The genomes of colistin-resistant *K. pneumoniae* isolates were used as the subject sequences. The identified mutations were analyzed using PROVEAN to predict their functional impact. The *mgrB* gene was PCR amplified as detailed in Supplementary Table 5, and the PCR products were sequenced by the Sanger method. The obtained sequences were mapped against the IS finder. If *mgrB* was not identified in the genome and PCR amplification with the previous primers was not successful, the absence of *mgrB* was corroborated through additional PCR amplification using the internal primers.

We used the module CONJscan of MacSyFinder to identify components of conjugation machinery. As this analysis was conducted using draft genomes the intention was not to detect complete conjugation systems or to classify them into the eight families, we only seek for presence or absence of such proteins. In addition, we perfomed the identification of relaxases (MOB) and plasmid replicons (Inc groups) using MOBscan and plasmidFinder (https://cge.food.dtu.dk/services/PlasmidFinder/), respectively.

The molecular epidemiology of twenty-six isolates and eleven reference strains was addressed by concatenating the sequence of the seven MLST genes obtained from https://bigsdb.pasteur.fr/klebsiella/ to construct a maximum-likelihood phylogenetic tree. iTOL v6 was used for visualizing and editing the tree^35^. The reference strains used in this study were: NTUH-K2044 (NZ_AB371288)^36^, SGH10 (NZ_CP025080)^37^, *K. pneumoniae* 2 (NJPL00000000) and 5 (NJPJ00000000)^25^, B8095 (NZ_MBSL00000000)^38^, KpPi144 (ONE205) (JAEDYN000000000)^39^, SB4935^14^, CG43^40^, TUM14036 (NZ_BIHP00000000)^6^, 10,271 (NZ_FKKF00000000) and 11,401 (JAUBZY000000000)^19^.

### Phylogenomic analysis

The assemblies for 37 isolates (26 isolates from this study and 11 reference strains) were used to construct a whole-genome SNP alignment using Snippy v.4.3.2 (https://github.com/ tseemann/snippy). A matrix of pairwise single nucleotide polymorphisms (SNPs) was then compiled with snp-dists v.0.7.0 (https://github.com/tseemann/snp-dists). *K. pneumoniae* SH10 (NC_000913.3) was set as reference. Construction of phylogenetic tree based on whole-genome SNP alignment was constructed with RAxML v.8 under the GTRGAMMA model. iTOL v6 was used to edit and visualized the tree.

### Statistical analysis

Survival curves were plotted using Kaplan–Meier were performed using GraphPad Prism 6 and Kaplan–Meier log rank and Wilcoxon-Gehan analysis. Correlation test was performed with ‘cor’ function using the Spearman rank correlation method from the ‘corrplot’ R package. Significant correlations were calculated with the ‘cor.test’ function and visualized with ‘corrplot’.

Virulence score was designated as follows, 1 = presence of core virulence genes *mrk*, *kfu*, *LPS*, and *fim*; 2 = core virulence genes and yersiniabactin; 3 = core virulence genes, yersiniabactin, and colibactin (or colibactin only); 4 = core virulence genes and aerobactin and/or salmochelin without yersiniabactin or colibactin; 5 = core virulence genes and aerobactin with yersiniabactin and salmochelin (no colibactin); 6 = core virulence genes and yersiniabactin, colibactin, salmochelin and aerobactin.

AMR score was designated as follows, 1 = intrinsic resistance to ampicillin due to chromosomal b-lactamase SHV or OKP; 2 = Acquired resistance to quinolone, aminoglycosides, tetracycline, sulfonamides, trimethoprim; 3 = traits from the category 2 and ESBL; 4 = traits from the category 2, carbapenemase and/or colistin resistance; 5 = traits from the category 2, ESBL or carbapenemase with colistin resistance; 6 = traits from the category 2 and ESBL with carbapenemase; 7 traits from the category 2 and ESBL, carbapenemase and colistin resistance.

### Ethics approval

This project was exempt from review by the Ethics Commission at National Institute of Public Health because it does not involve human subjects and/or it is not an academic study and/or it does not include the analysis of data previously obtained from another study requiring the patients’ informed consent. On the other hand, the clinical isolates included in the study were obtained by routine procedures in each of the hospitals involved.

### Supplementary Information

Supplementary Information 1.

Supplementary Information 2.

## Data Availability

The accession number of the strains from this study were deposited under the BioProject PRJNA985271.
