# Supplementary material for: Comprehensive study reveals phenotypic heterogeneity in Klebsiella pneumoniae species complex isolates
Source: Sci Rep. 2024 Mar 11;14:5876. doi: 10.1038/s41598-024-55546-z (PMC10928225; doi:10.1038/s41598-024-55546-z)
Supplement: Supplementary file 1 — Supplementary Information 1. [file 41598_2024_55546_MOESM1_ESM.pptx]

## Slide 1
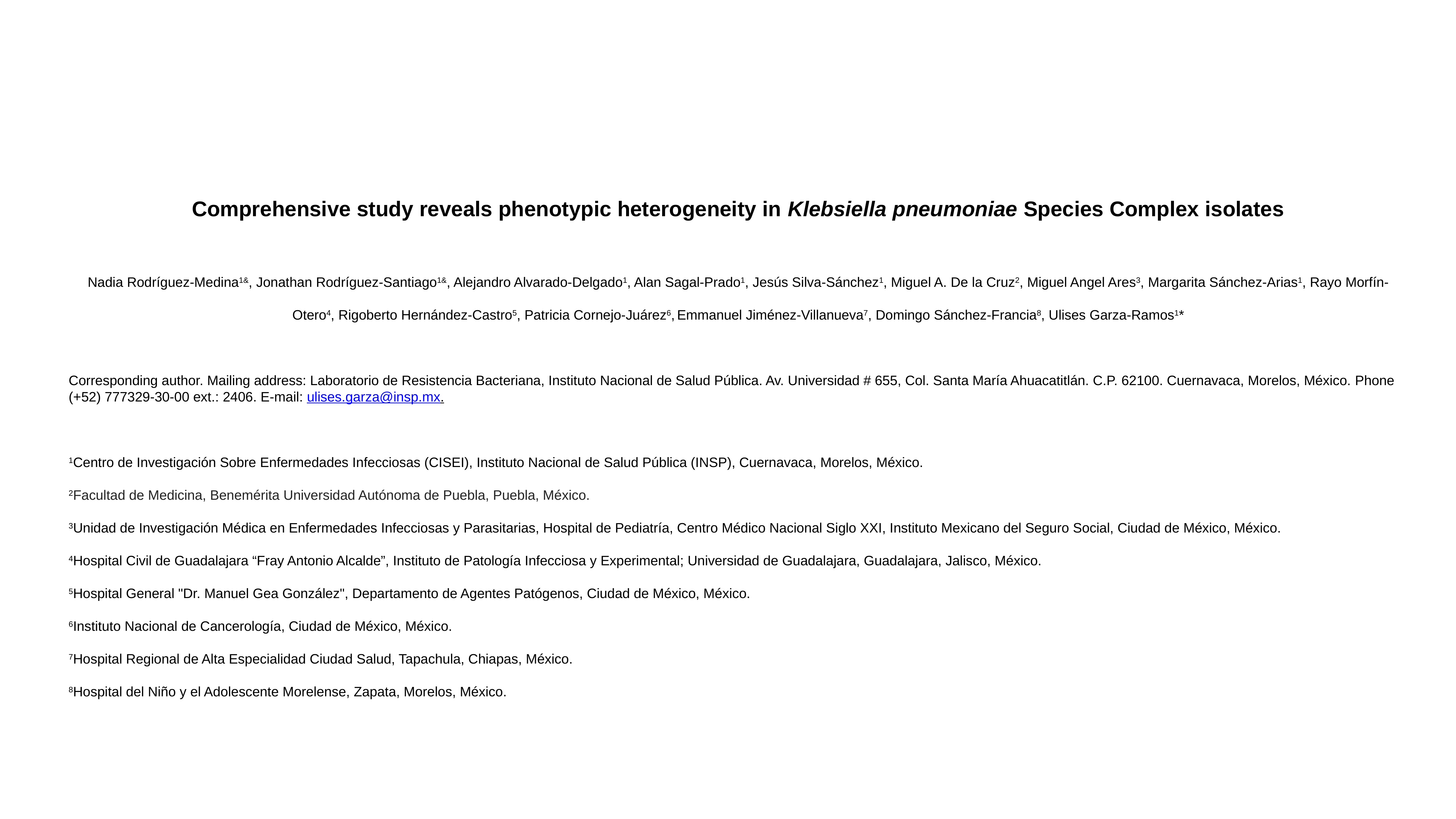

Comprehensive study reveals phenotypic heterogeneity in Klebsiella pneumoniae Species Complex isolates
Nadia Rodríguez-Medina1&, Jonathan Rodríguez-Santiago1&, Alejandro Alvarado-Delgado1, Alan Sagal-Prado1, Jesús Silva-Sánchez1, Miguel A. De la Cruz2, Miguel Angel Ares3, Margarita Sánchez-Arias1, Rayo Morfín-Otero4, Rigoberto Hernández-Castro5, Patricia Cornejo-Juárez6, Emmanuel Jiménez-Villanueva7, Domingo Sánchez-Francia8, Ulises Garza-Ramos1*
Corresponding author. Mailing address: Laboratorio de Resistencia Bacteriana, Instituto Nacional de Salud Pública. Av. Universidad # 655, Col. Santa María Ahuacatitlán. C.P. 62100. Cuernavaca, Morelos, México. Phone (+52) 777329-30-00 ext.: 2406. E-mail: ulises.garza@insp.mx.
1Centro de Investigación Sobre Enfermedades Infecciosas (CISEI), Instituto Nacional de Salud Pública (INSP), Cuernavaca, Morelos, México.
2Facultad de Medicina, Benemérita Universidad Autónoma de Puebla, Puebla, México.
3Unidad de Investigación Médica en Enfermedades Infecciosas y Parasitarias, Hospital de Pediatría, Centro Médico Nacional Siglo XXI, Instituto Mexicano del Seguro Social, Ciudad de México, México.
4Hospital Civil de Guadalajara “Fray Antonio Alcalde”, Instituto de Patología Infecciosa y Experimental; Universidad de Guadalajara, Guadalajara, Jalisco, México.
5Hospital General "Dr. Manuel Gea González", Departamento de Agentes Patógenos, Ciudad de México, México.
6Instituto Nacional de Cancerología, Ciudad de México, México.
7Hospital Regional de Alta Especialidad Ciudad Salud, Tapachula, Chiapas, México.
8Hospital del Niño y el Adolescente Morelense, Zapata, Morelos, México.

## Slide 2
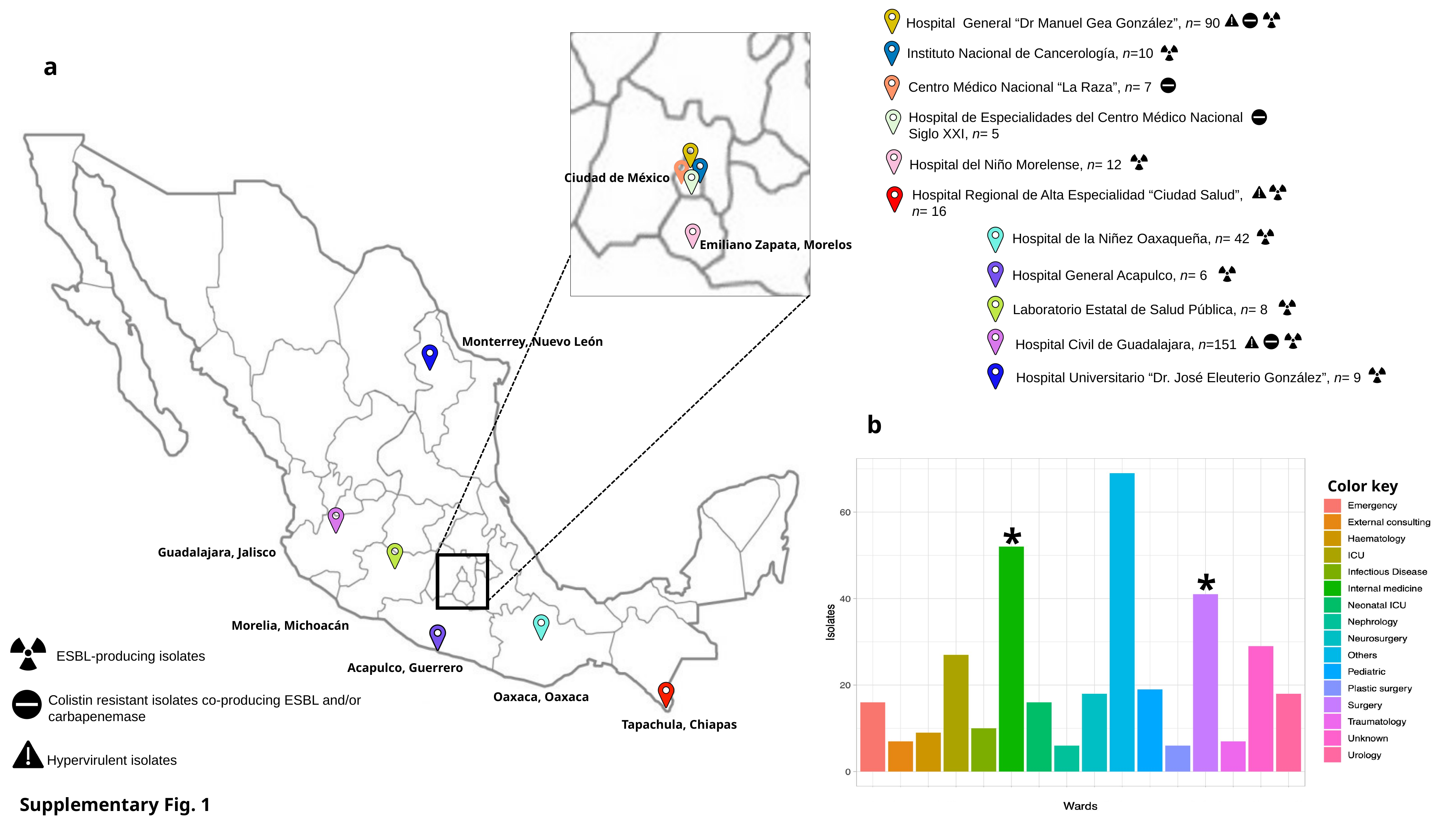

Hospital General “Dr Manuel Gea González”, n= 90
Instituto Nacional de Cancerología, n=10
a
Centro Médico Nacional “La Raza”, n= 7
Hospital del Niño Morelense, n= 12
Ciudad de México
Hospital Regional de Alta Especialidad “Ciudad Salud”, n= 16
Hospital de la Niñez Oaxaqueña, n= 42
Emiliano Zapata, Morelos
Hospital General Acapulco, n= 6
Laboratorio Estatal de Salud Pública, n= 8
Monterrey, Nuevo León
Hospital Civil de Guadalajara, n=151
Hospital Universitario “Dr. José Eleuterio González”, n= 9
b
Guadalajara, Jalisco
Morelia, Michoacán
Acapulco, Guerrero
Oaxaca, Oaxaca
Tapachula, Chiapas
Hospital de Especialidades del Centro Médico Nacional Siglo XXI, n= 5
Color key
*
*
ESBL-producing isolates
Colistin resistant isolates co-producing ESBL and/or carbapenemase
Hypervirulent isolates
Supplementary Fig. 1

## Slide 3
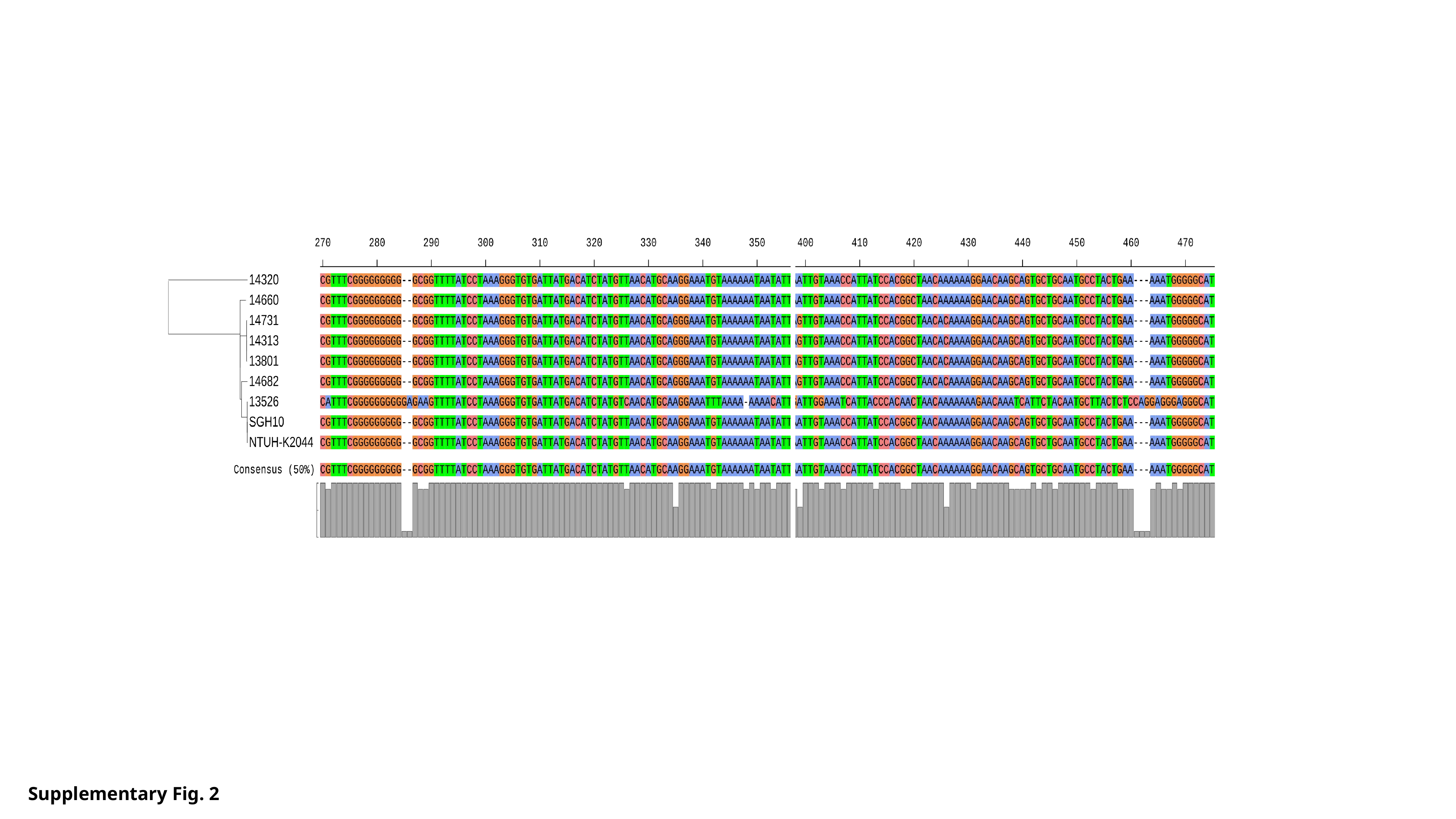

Supplementary Fig. 2
