# Supplementary material for: Comprehensive study reveals phenotypic heterogeneity in Klebsiella pneumoniae species complex isolates
Source: Sci Rep. 2024 Mar 11;14:5876. doi: 10.1038/s41598-024-55546-z (PMC10928225; doi:10.1038/s41598-024-55546-z)
Supplement: Supplementary file 2 — Supplementary Information 2. [file 41598_2024_55546_MOESM2_ESM.docx]

**Supplementary Table 1**. Characteristics of classical colistin-resistant *K. pneumoniae* isolates.

| **Isolate ID** | **Genotyping**  **Clone^a^** | **Hospital** | **MIC (mg/L)** | | **Carbapenemase** | **ESBL** | **Chromosomal mutations^b^** | | | | |
| --- | --- | --- | --- | --- | --- | --- | --- | --- | --- | --- | --- |
|  |  |  | **Colistin** | **Imipenem** |  |  | **MgrB**  **(47aa)** | **CrrB**  **(353 aa)** | **PmrA**  **(223 aa)** | **PmrB**  **(365 aa)** | **PhoQ**  **(488 aa)** |
| 7040 | A | 1 | 16 | 64 | NDM-1 | CTX-M-15 | Disruption by IS1X2  (family IS1) | Wt | Wt | R256G^c^ | Wt |
| 14669 | A | 2 | 32 | >64 | NDM-1 | CTX-M-15 | Disruption by IS1X2  (family IS1) | Wt | Wt | R256G^c^ | Wt |
| 13861 | A | 3 | 32 | >64 | NDM-1 | CTX-M-15 | Disruption by ISKpn26  (family IS5) | Wt | Wt | R256G^c^ | Wt |
| 13863 | A | 3 | 32 | 32 | NDM-1 | CTX-M-15 | Wt | Wt | Wt | R256G^c^ | Wt |
| 13862 | A | 3 | 16 | >64 | NDM-1 | CTX-M-15 | Frameshift at K3 | Wt | Wt | R256G^c^ | Wt |
| 7034 | NR | 1 | 32 | >64 | KPC-2 | - | Disruption by ISKpn18  (family IS3) | Wt | Wt | R256G^c^ | Wt |
| 13193 | NR | 3 | 8 | 16 | - | CTX-M-15 | C28R | Wt | Wt | Wt | Wt |
| 6296 | NR | 4 | 32 | >64 | OXA-232 | - | Disruption by IS903  (family IS5) | Missing | Wt | R256G^c^ | Wt |
| 13576 | NR | 2 | 32 | >64 | OXA-232 | CTX-M-15 | Complete deletion | Wt | Wt | Wt | Wt |
| 14657 | NR | 2 | 8 | 4 | - | CTX-M-15 | Wt | Missing | G53C | Wt | L348Q |
| 5166 | NR | 1 | 4 | 0.5 | - | - | Wt | Wt | Wt | R256G^c^ | Wt |

^a^These colistin-resistant *K. pneumoniae* isolates were clonally related (clone A) and identified on three different hospitals.

^b^Only mutations with predicted functional impact using the PROVEAN software are shown.

^c^It has been experimentally ruled out that the R256G mutation in the PmrB protein is responsible for colistin resistance.

Hospitals: 1, Centro Médico Nacional “La Raza”; 2, Hospital General “Dr. Manuel Gea González”; 3, Hospital Civil de Guadalajara; 4, Centro Médico Nacional “Siglo XXI”.

NR = No related; - = negative; WT, Wild-type.

**Supplementary Table 2.** Clinical characteristics of presumptive hypervirulent and hypermucoviscous-like KpSC isolates.

| **Strain** | **Bacterial specie** | **Phenotype** | **MLST** | **Hospital** | **Sex** | **Age** | **Origin** | **Infection type/source** |
| --- | --- | --- | --- | --- | --- | --- | --- | --- |
| 14313 | *K. pneumoniae* | p-hv | ST3999 | Hospital Regional de Alta Especialidad “Ciudad Salud” | Male | 73 | HAI | Wound infection |
| 14731 | *K. pneumoniae* | p-hv | ST3999 | Hospital GEA Gonzalez | Female | Missing | CAI | Pneumonia |
| 14660 | *K. pneumoniae* | p-hv | ST86 | Hospital GEA Gonzalez | Female | 56 | CAI | Ascites |
| 13801 | *K. pneumoniae* | p-hv | ST3999 | Hospital Civil de Guadalajara | Male | Missing | CAI | Eye secretion |
| 14682 | *K. pneumoniae* | p-hv | ST380 | Hospital GEA Gonzalez | Female | 69 | CAI | UTI |
| 13526 | *K. pneumoniae* | p-hv | ST23 | Hospital GEA Gonzalez | Female | 71 | CAI | UTI |
| 14320* | *K. quasipneumoniae* | p-hv | ST3857 | Hospital Regional de Alta Especialidad “Ciudad Salud” | Male | 88 | Missing | Pneumonia |
| 14636 | *K. pneumoniae* | hmv-like | ST45 | Hospital GEA Gonzalez | Male | 76 | CAI | UTI |
| 3478 | *K. pneumoniae* | hmv-like | ST530 | Hospital del Niño Morelense | Female | 8 | CAI | UTI |
| 14346 | *K. pneumoniae* | hmv-like | ST307 | Hospital Regional de Alta Especialidad "Ciudad Salud” | Male | 44 | Missing | BSI |
| 14671 | *K. pneumoniae* | hmv-like | ST469 | Hospital GEA Gonzalez | Female | 84 | CAI | UTI |
|  |  |  |  |  |  |  |  |  |
|  |  |  |  |  |  |  |  |  |

HAI: Hospital-acquired Infection; CAI: Community-acquired Infection; p-hv, presumptive hypervirulent.

**K. quasipneumoniae* subsp. *similipneumoniae*

**Supplementary Table 3**. Detailed information of pathogenicity determination, virulome and resistome of selected KpSC isolates*.*

| **Isolate** | **Bacterial Species** | **Source** | **PFGE** | **Classification** | **Capsular type** | **ST** | ***In vivo* and *in vitro* assays** | | | | |  |  | **Genomic analysis** | |
| --- | --- | --- | --- | --- | --- | --- | --- | --- | --- | --- | --- | --- | --- | --- | --- |
|  |  |  |  |  |  |  | **Mortality rate % (CFU)** | **Sedimentation assay (OD_600_)** | **Uronic**  **acid (μg/10^9^CFU)** | **Serum resistance** | **Phagocytosis (CFU/ml)** | **Biofilm index** |  | **β-lactam**  **Resistome** | **Virulome** |
| 13526 | *K. pneumoniae* | Urine | NR | atypical hv | KL1 | 23 | 100 (10^8^) | 0.293 | 35.1121 | Resistant | 4833 | 0.5 |  | SHV-190 | *mrkABDFIJ, fimA, ybtAEPSTX irp2, iucABCD iutA, iroBCN, entABCDEF, rmpA, rmpA2, allABCDRS, arcC, glcKR, hyi, mceABCDEGHIJ, clbACDEFGILOPQ, kfuABC, fyuA* |
| 14320 | *K. quasipneumoniae* subsp. *similipneumoniae* | Sputum | NR | hv | KL1 | No applied | 100 (10^8^) | 0.687 | 64.7758 | Resistant | 180 | 0.2 |  | OKP-B | *mrkABDFIJ, fimA, iucABCD iutA, iroBCN, entABCDEF, rmpA, rmpA2* |
| 14313 | *K. pneumoniae* | Wound secretion | NR | hv | KL2 | 3999 | 100 (10^8^) | 0.850 | 73.3756 | Resistant | 2100 | 0.6 |  | SHV-11 | *mrkABDFIJ, fimA, ybtAEPSTX irp2, iucABCD iutA, iroBCN, entABCDEF, kfuABC* |
| 14660 | *K. pneumoniae* | Ascites | NR | hv | KL2 | 86 | 100 (10^2^) | 0.667 | 54.4843 | Resistant | 571 | 0.5 |  | SHV-1 | *mrkABDFIJ, fimA,* |
| 14731 | *K. pneumoniae* | Sputum | NR | hv | KL2 | 3999 | 100 (10^8^) | 0.423 | 67.6366 | Resistant | 4866 | 0.7 |  | SHV-11 | *mrkABDFIJ, fimA, ybtAEPSTX irp2, iucABCD iutA, iroBCN, entABCDEF, kfuABC* |
| 14682 | *K. pneumoniae* | Urine | NR | atypical hv | KL2 | 380 | 100 (10^8^) | 0.286 | 21.7937 | Resistant | 4750 | 0.2 |  | SHV-33 | *mrkABDFIJ, fimA, ybtAEPSTX irp2, iucABCD iutA, iroBCN, entABCDEF, rmpA, mceABCDEGHIJ, kfuABC, fyuA* |
| 13801 | *K. pneumoniae* | Eye secretion | NR | atypical hv | KL2 | 3999 | 100 (10^8^) | 0.235 | 33.3997 | Sensitive | 5876 | 0.3 |  | SHV-11 | *mrkABDFIJ, fimA, ybtAEPSTX irp2, iucABCD iutA, iroBCN, entABCDEF, kvgA,rmpA* |
| 3478 | *K. pneumoniae* | Urine | NR | hmv-like | KL54 | 530 | 60 (3x10^8^) | 0.507 | 59.3274 | Resistant | 4306 | 0.5 |  | SHV-1, TEM-1, CTX-M-14 | *mrkABDFIJ, fimA, iutA, kfuABC* |
| 14346 | *K. pneumoniae* | Blood | NR | hmv-like | KL102 | 307 | 100 (3x10^8^) | 0.354 | 24.8206 | Sensitive | 9930 | 0.5 |  | SHV-28, TEM-1, CTX-M-15, OXA-1 | *mrkABDFIJ, fimA, iutA, kfuABC* |
| 14636 | *K. pneumoniae* | Urine | NR | hmv-like | KL24 | 45 | 100 (3x10^8^) | 0.808 | 145.8969 | Sensitive | 584 | 0.8 |  | SHV-1, TEM-1, CTX-M-15 | *mrkABDFIJ, fimA, ybtAEPSTX irp2, fyuA, kfuABC* |
| 14671 | *K. pneumoniae* | Urine | NR | hmv-like | KL139 | 469 | 100 (3x10^8^) | 0.421 | 38.7444 | Sensitive | 7546 | 0.4 |  | SHV-11 | *mrkABDFIJ, fimA, iutA, kfuABC* |
| 7040 | *K. pneumoniae***^a^** | Blood | A | cl | KL27 | 392 | 0 (10^8^) | 0.129 | 2.4215 | Sensitive | 825,000 | 0.2 |  | SHV-67, TEM-1, CTX-M-15, OXA-1, OXA-232, NDM-1 | *mrkABDFIJ, fimA,entB, kfuABC* |
| 13861 | *K. pneumoniae***^a^** | Urine | A | cl | KL27 | 392 | 0 (10^8^) | 0.125 | ND | ND | ND | ND |  | SHV-67, TEM-1, CTX-M-15, OXA-1, NDM-1 | *mrkABDFIJ, fimA, entB, kfuABC* |
| 13862 | *K. pneumoniae***^a^** | Secretion | A | cl | KL27 | 392 | 0 (10^8^) | 0.122 | 12.9148 | Sensitive | 508,333 | 0.1 |  | SHV-67, TEM-1, CTX-M-15, OXA-1, NDM-1 | *mrkABDFIJ, fimA, entB, kfuABC* |
| 13863 | *K. pneumoniae***^a^** | Secretion | A | cl | KL27 | 392 | 0 (10^8^) | 0.103 | 10.4933 | Sensitive | 844666 | 0.3 |  | SHV-67, TEM-1, CTX-M-15, OXA-1, NDM-1 | *mrkABDFIJ, fimA, entB, kfuABC* |
| 14669 | *K. pneumoniae***^a^** | Urine | A | cl | KL27 | 392 | 0 (10^8^) | 0.144 | 4.4395 | Sensitive | 2873333 | 0.1 |  | SHV-67, TEM-1, CTX-M-15, OXA-1, NDM-1 | *mrkABDFIJ, fimA, entB, kfuABC* |
| 5166 | *K. pneumoniae***^a^** | Urine | NR | cl | KL15 | 340 | 30 (10^8^) | 0.211 | 3.0269 | Sensitive | 1050000 | 1.0 |  | SHV-182, TEM-1 | *mrkABDFIJ, fimA, entB, kfuABC* |
| 7034 | *K. pneumoniae***^a^** | Blood | NR | cl | KL74 | 258 | 0 (10^8^) | 0.278 | 5.852 | Sensitive | 825000 | 0.5 |  | SHV-12,  OXA-9, KPC-2 | *mrkABDFIJ, fimA, entB, kfuABC* |
| 14657 | *K. pneumoniae***^a^** | Urine | NR | cl | KL12 | 3998 | ND | 0.179 | 3.2287 | Sensitive | 1523333 | 0.02 |  | SHV-44, TEM-1, CTX-M-15 | *mrkABDFIJ, fimA, entB, kfuABC* |
| 13193 | *K. pneumoniae***^a^** | Urine | NR | cl | KL102 | 3227 | ND | 0.239 | 8.0717 | Sensitive | 21333 | 0.7 |  | SHV-28, TEM-1, CTX-M-15 | *mrkABDFIJ, fimA,entB, kfuABC* |
| 6296 | *K. pneumoniae***^a^** | Sputum | NR | cl | KL24 | 45 | ND | 0.195 | ND | ND | ND | ND |  | SHV-1, OXA-232 | *mrkABDFIJ, fimA,entB, kfuABC* |
| 13576 | *K. pneumoniae***^a^** | Secretion | NR | cl | KL58 | 1017 | ND | 0.132 | ND | ND | ND | ND |  | SHV-11, CTX-M-15, OXA-232 | *mrkABDFIJ, fimA,entB, kfuABC* |
| 9459 | *K. pneumoniae* | Blood | NR | cl | KL23 | 280 | ND | 0.175 | ND | ND | ND | ND |  | SHV-28, TEM-1, CTX-M-15 | *mrkABDFIJ, fimA, entB, kfuABC* |
| 11271 | *K. pneumoniae* | Blood | NR | cl | KL24 | 15 | 0 (10^8^) | 0.133 | 7.46 | Sensitive | 31533 | 0.6 |  | SHV-28, TEM-1, CTX-M-15 | *mrkABDFIJ, fimA, ybtAEPSTX irp2, kfuABC, fyuA* |
| 11273 | *K. pneumoniae* | Blood | NR | cl | KL127 | 45 | ND | 0.143 | ND | ND | ND | ND |  | SHV-1, TEM-1, CTX-M-15 | *mrkABDFIJ, fimA, ybtAEPSTX irp2, fyuA* |
| 21-3274 | *K. pneumoniae* | Urine | NR | cl | KL2 | 25 | ND | 0.247 | 8.2735 | Resistant | 832000 | 0.3 |  | SHV-11, TEM-1, CTX-M-15 | *mrkABDFIJ, fimA, ybtAEPSTX irp2, fyuA* |

^a^These correspond to colistin-resistant isolates. Clone A corresponds to clonally related colistin-resistant *K. pneumoniae* isolates; for more details see Supplementary Table 1.

Abbreviations: NR, not related; ND, Not determined.

**Supplementary Table 4**. Presence of conjugative machinery proteins and identification of relaxase family of KpSC isolates and reference strains.

| **Isolate** | **Classification** | **Presence of conjugation components** | **Relaxase family** |
| --- | --- | --- | --- |
| 13526 | atypical hv | Yes | MOBH |
| 14320* | hv | No | MOBH |
| 14313 | hv | Yes | MOBF, MOBC |
| 14660 | hv | Yes | MOBH, MOBF, MOBC |
| 14682 | atypical hv | Yes | MOBF, MOBC, MOBH, MOBH |
| 14731 | hv | Yes | MOBC, MOBF |
| 13801 | atypical hv | Yes | MOBF, MOBC |
| 3478 | hmv-like | Yes | MOBP, MOBF |
| 14346 | hmv-like | Yes | MOBF, MOBP |
| 14636 | hmv-like | Yes | MOBF, MOBC, MOBP |
| 14671 | hmv-like | No | No relaxase found |
| 7040 | cl | Yes | MOBF, MOBF |
| 13861 | cl | Yes | MOBF, MOBF, MOBH |
| 13862 | cl | Yes | MOBF, MOBF, MOBH |
| 13863 | cl | Yes | MOBF, MOBF, MOBH |
| 14669 | cl | Yes | MOBF, MOBF |
| 5166 | cl | Yes | MOBP, MOBF, MOBH |
| 7034 | cl | Yes | MOBC, MOBQ, MOBH, MOBP, MOBC |
| 9459 | cl | Yes | MOBF, MOBP |
| 11271 | cl | Yes | MOBC, MOBF |
| 11273 | cl | Yes | MOBC, MOBQ, MOBP, MOBF |
| 13193 | cl | Yes | MOBH, MOBH, MOBP, MOBF |
| 14657 | cl | Yes | MOBF, MOBP |
| 21-3274 | cl | Yes | MOBC, MOBF |
|  | |  |  |
| 10271 | hv | No | No relaxase found |
| 11401 | hv | Yes | MOBH, MOBF, MOBC |
| SGH10 | hv | Yes | MOBC, MOBH |
| NTIH-K2044 | hv | Yes | MOBC, MOBH |
| CG43 | hv | No | MOBH |
| TUM14036 | hv | Yes | MOBC, MOBH |
| SB4935** | hv | Yes | MOBC |
| B8095* | hv | Yes | MOBC, MOBH |

**K. quasipneumoniae* subsp. *similipneumoniae*

***K. quasipneumoniae* subsp*. quasipneumoniae*

**Supplementary Table 5**. Marker genes and capsular types screened in the KpSC isolates.

| **Test, gene, and capsular type** | **Oligonucleotides**  **(5’ - 3’)** | **References** | **Phenotype** | | |
| --- | --- | --- | --- | --- | --- |
|  |  |  | **Classical** | **Hypervirulent** | **Hypermucoviscous-like** |
| String test | Not applied | 1 | - | +/- | + |
| *rmpA* | rmpA-F (ACTGGGCTACCTCTGCTTCA)  rmpA-R (CTTGCATGAGCCATCTTTCA) | 2 | - | + | - |
| *rmpA2* | rmpA2-F (CTTTATGTGCAATAAGGATGTT)  rmpA2-R (CCTCCTGGAGAGTAAGCATT) | 2 | - | +/- | - |
| *iucA* | iucA-F (AATCAATGGCTATTCCCGCTG)  iucA-R (CGCTTCACTTCTTTCACTGACAGG) | 3 | - | + | - |
| *irp2* | irp2-F (GCTACAATGGGACAGCAACGAC)  irp2-R (GCAGAGCGATACGGAAAATGC) | 3 | +/- | +/- | +/- |
| K1 | K1-F (GGTGCTCTTTACATCATTGC)  K1-R (GCAATGGCCATTTGCGTTAG) | 4 | - | +/- | - |
| K2 | K2-F (GACCCGATATTCATACTTGACAGAG)  K2-R (CCTGAAGTAAAATCGTAAATAGATGGC) | 5 | +/- | +/- | - |
| *mgrB* | mgrB_ext_F (AAGGCGTTCATTCTACCACC)  mgrB_ext_R (TTAAGAAGGCCGTGCTATCC) | 6 | + | - | - |

Symbols: +, presence; -, absence; +/-; could be positive or negative.

1. Hadano Y. String test. *BMJ Case Rep*. **15**, bcr2012008328 (2013).
2. Lee CH, et al. Hypermucoviscosity associated with Klebsiella pneumoniae-mediated invasive syndrome: a prospective cross-sectional study in Taiwan. *Int J Infect Dis*. **14**, e688-92 (2010).
3. Russo, T.A. et al. Aerobactin mediates virulence and accounts for increased siderophore production under iron-limiting conditions by hypervirulent (hypermucoviscous) Klebsiella pneumoniae. *Infection and immunity*. **82**, 2356-67 (2014).
4. Pan, Y.J. et al. Capsular types of Klebsiella pneumoniae revisited by wzc sequencing. *PloS one*. **8**, e80670 (2013).
5. Turton, J.F, et al. PCR characterization and typing of Klebsiella pneumoniae using capsular type-specific, variable number tandem repeat and virulence gene targets. *J. Med. Microbiol*. **59**, 541-7 (2010).
6. Cannatelli, A. et al. MgrB inactivation is a common mechanism of colistin resistance in KPC-producing *Klebsiella pneumoniae* of clinical origin. Antimicrob Agents Chemother. **58**, 5696-703 (2014).
